# Endophthalmitis following photorefractive keratectomy with a history of radial keratotomy: a case report

**DOI:** 10.1186/1869-5760-3-31

**Published:** 2013-02-11

**Authors:** Peter A Karth, John W Karth

**Affiliations:** 1 Department of Ophthalmology, Medical College of Wisconsin, 1918 E. Lafayette Pl., Unit 507, 53202, Milwaukee, WI, USA; 2 Oregon Eye Associates, 1550 Oak Street Suite 7, 97401, Eugene, OR, USA

**Keywords:** Endophthalmitis, Radial keratotomy, Photorefractive keratectomy, Endophthalmitis without keratitis, Methicillin-resistant *Staphylococcus aureus*

## Abstract

**Background:**

We report the case of an 81-year-old woman with a history of radial keratotomy 9 years prior who developed endophthalmitis without preceding keratitis 4 days after uneventful photorefractive keratectomy surgery. This case report utilized clinical examination and microbacterial laboratory findings.

**Findings:**

Diagnosis of bacterial endophthalmitis was made via clinical examination and cultured vitreous tap which grew methicillin-resistant *Staphylococcus aureus*. No preceding keratitis was seen on exam. The patient responded to intravitreal antibiotics.

**Conclusions:**

We believe that the epithelium removed during the photorefractive keratectomy procedure may have uncovered areas of full-thickness radial keratotomy incisions allowing bacterial ingress, causing bacterial endophthalmitis without preceding keratitis.

## Findings

### Introduction

Radial keratotomy (RK) is a procedure in which multiple radial incisions are made in the corneal epithelium and stroma, ideally reaching a depth of 85% to 95% of the total cornea thickness for the purposes of reducing or eliminating refractive error. Photorefractive keratectomy (PRK) involves the removal of the corneal epithelium to expose the stroma for refractive laser photoablation. We report a case of postsurgical endophthalmitis following photorefractive keratectomy in a patient with a remote history of radial keratotomy.

### Case report

In 2009, an 81-year-old woman underwent unilateral myopic PRK in the left eye with the goal of emmetropia. Preoperatively, her Snellen best-corrected visual acuity (BCVA) was 20/25 with a refractive error of −0.75 spherical equivalent in the left eye. She had significant past ocular history of uncomplicated eight-incision RK procedure (without arcuate keratotomy) in the left eye 9 years previously and uneventful cataract surgery 10 years previously in the left eye with temporal clear corneal incision and posterior chamber intraocular lens implantation. For the PRK procedure, the epithelium was removed by alcohol-assisted (20% ethanol solution) de-epithelialization; no mitomycin C was used. The procedure had no intraoperative complications per report.

On the first postoperative day following PRK in the left eye, the patient's exam showed normal postsurgical findings and no signs of infection; a large epithelial defect was noted as expected. Her BCVA was 20/50 with a bandage contact lens. Ophthalmic medications were nepafenac ophthalmic suspension 0.1% three times per day and tobramycin 0.3%/dexamethasone 0.1% four times per day.

On the fourth postoperative day, the patient began to experience symptoms of blurry vision, redness, and irritation. She did not seek care until the sixth postoperative day, when she presented with redness, irritation, and decreased vision in the left eye. The patient's visual acuity was 20/400. Examination showed a 5-mm central corneal epithelial defect, corneal edema, and 10% hypopyon in the anterior chamber. White cells were seen in the anterior vitreous. No corneal infiltrate was noted, and RK incisions were not noted to be gaping. No gross corneal ectasia and other abnormal corneal features were noted; the cataract surgery wound was not noted to be abnormal. Topical drops were begun: prednisolone acetate 1% ophthalmic suspension and moxifloxacin ophthalmic every 30 min.

On the next day, she developed worsening left eye pain and continued severe injection. Visual acuity was counting fingers. There was no view to the posterior pole due to corneal edema; the anterior chamber hypopyon remained at 10%. There was concern for endophthalmitis at this time, and a vitreoretinal consultation was obtained. Extensive vitreous debris was seen on B-scan ultrasound. Postoperative endophthalmitis was diagnosed. A vitreous tap was performed, and the aspirate was sent for culture. Intravitreal injection of vancomycin and ceftazidime was performed. The patient was continued on topical antibiotics drops as previously dosed.

From postoperative day 8, the patient was seen regularly and general improvement ensued with decreased anterior and posterior segment cells and resolving hypopyon. The cultures of the vitreous aspirate yielded methicillin-resistant *Staphylococcus aureus* sensitive to vancomycin. The patient's vision improved, and signs and symptoms of endophthalmitis resolved. The hypopyon diminished and the cornea edema cleared over the next 3 weeks. At the last follow-up, her best-corrected visual acuity was 20/100.

### Discussion

In this case, we present a patient with a history of RK 9 years prior who underwent uncomplicated PRK and then developed postoperative bacterial endophthalmitis. We believe that the removal of corneal epithelium as part of the PRK procedure may have re-opened one or more full-thickness RK incisions, allowing egress of bacteria into the anterior chamber. While RK incisions typically extend through 85% to 95% of the corneal stroma, it is common for incisions to transverse the entire thickness of the cornea and cause microperforations. Typically after a RK procedure, the superficial cornea re-epithelializes, covering RK incisions and sealing off the incisions from possible inoculums. PRK requires the removal of the corneal epithelia prior to the stromal photoablation, thus removing the epithelial layer protecting these full-thickness RK incisions. We believe that this breach allowed bacterial inoculum ingress to the anterior chamber, causing bacterial endophthalmitis.

Microperforation is a known occurrence in radial keratotomy surgery. In a study of 466 radial keratotomy procedures, the rate of microperforation was found to be 3.8% with additional larger perforations [1]. The rate of microperforations could vary based on the individual surgeon. Typically, the corneal epithelium quickly heals and covers these incisions, preventing an open path to the anterior chamber.

In Jain and Azar's review of the literature, 43 cases of infectious endophthalmitis were reported after RK surgery; 47% occurred in the first 2 weeks after the procedure [2]. Late-occurring bacterial keratitis after radial keratotomy or astigmatic keratotomy with some cases progressing to endophthalmitis has been reported [3,4]. Infectious keratitis after RK is thought to occur due to corneal epithelial breakdown in the setting of dry eye syndrome or infectious corneal ulcers and may occur years after the original RK procedure. Bacterial endophthalmitis without preceding keratitis has also been reported shortly after enhancement to a previous RK procedure [5]. In 2004, a comprehensive review of the literature by Chang, et al. yielded three eyes with a history of RK which were found to have bacterial keratitis following laser *in situ* keratomileusis; they also reported another eye with a history of RK and PRK that was found to have bacterial keratitis following laser *in situ* keratomileusis [6]. On our review, no published cases of endophthalmitis without keratitis following PRK in patients with a history of RK were found.

In our case, no corneal keratitis was noted. We feel that uncovering microperforations from the RK surgery during the PRK de-epithelialization may have allowed for direct inoculation of the anterior chamber leading to endophthalmitis, rather than migration of a primarily corneal infection to the rest of the eye as seen in cases with preceding keratitis.

This case demonstrates possible severe complications associated with uncovering microperforations in epithelia-altering procedures in patients with a history of RK even many years after the RK procedure.

## Competing interest

The authors declare that they have no competing interest.

## Authors’ contributions

JK identified the case as reportable. PK and JK designed the chart review. PK carried out the chart review. PK wrote the manuscript draft, and PK/JK edited the final manuscript. Both authors read and approved the final manuscript.

## References

[B1] Leroux les Jardins S Bertrand I Massin M Intraoperative and early postoperative complications in 466 radial keratotomies Refract Corneal Surg 1992 3 3 215 216 1633140

[B2] Jain S Azar DT Eye infections after refractive keratotomy J Refract Surg 1996 3 1 148 155 8963804 10.3928/1081-597X-19960101-25

[B3] Erkin EF Durak I Ferliel S Maden A Keratitis complicated by endophthalmitis 3 years after astigmatic keratotomy J Cataract Refract Surg 1998 3 9 1280 1282 10.1016/S0886-3350(98)80029-1 9768410

[B4] Levy J Hirsh A Klemperer I Lifshitz T Late-onset Pseudomonas keratitis after radial keratotomy and subsequent laser in situ keratomileusis: case report and literature review Can J Ophthalmol 2005 3 2 211 213 16049542 10.1016/S0008-4182(05)80038-9

[B5] Heidemann DG Dunn SP Haimann M Endophthalmitis after radial keratotomy enhancement J Cataract Refract Surg 1997 3 6 951 953 10.1016/S0886-3350(97)80259-3 9292684

[B6] Chang MA Jain S Azar DT Infections following laser in situ keratomileusis: an integration of the published literature Surv Ophthalmol 2004 3 3 269 280 10.1016/j.survophthal.2004.02.007 15110665

